# Temporal Framework and Biological Indicators of Non-Suicidal Self-Injury and Related Behaviours

**DOI:** 10.21315/mjms2024.31.4.18

**Published:** 2024-08-27

**Authors:** Uzma Ilyas, Ayera Butt, Khurram Awan, Javeria Asim, Muhammad Suleman Shakoor, Muqadas Fatima

**Affiliations:** 1 Department of Psychology, University of Central Punjab, Lahore, Pakistan; 2 Department of Psychology, Lahore Garrison University, DHA, Lahore, Pakistan; 3 Pakistan Institute of Learning and Living, Lahore, Pakistan

**Keywords:** impulsivity, non-suicidal self-injury (NSSI), anatomy, neurotransmitters, biochemistry

## Abstract

Adolescence is a transitional stage between puberty and maturity. Significant alterations in brain chemistry and hormone activity cause mood swings and other psychological and physical symptoms. On their journey to adolescence, adolescents deal with complex emotions, moral dilemmas, sexual concerns, identity crises and particular societal expectations related to their upbringing. Impulsivity in adolescents is frequent and causes multiple issues. Impulsivity often lead towards non-suicidal self-injury (NSSI), which has devastating consequences, which are both physical and mental. Both impulsivity and NSSI have their roots in brain chemistry and its related functions. The aim of this special communication was to delve into brain chemistry through studying the function of neurotransmitters and brain areas in NSSI and impulsivity. Multiple papers were sought on the topic of neurochemistry and neuroanatomy. The results identified serotonin, dopamine and glutamate as the neurotransmitters responsible for NSSI and impulsivity. Dysregulation in these neurotransmitters lead to the presentation of NSSI and impulsivity. Other than the neurotransmitters, the brain areas identified were prefrontal cortex, medial prefrontal cortex and ventrolateral prefrontal cortex. The compiled results of this research would help individuals in understanding the neurotransmitters and the brain areas responsible. This would also help in generating awareness regarding the biological nature of the phenomenon as well, leading to less stigmatisation. The less stigmatisation towards these phenomena can help the affected individuals to seek help without any guilt or shame, along with support from society as well.

## Introduction

There are a lot of changes during adolescence both physically and mentally which encompasses identity exploration, and forming of relationships (1). However, it is not without challenges, such as mood swings attributed to hormonal changes (2) along with development of moral and ethical frameworks (3). Adolescence is a time of significant cognitive and emotional development for individuals, along with possible challenges like physical changes, gender stereotypes, and discrimination based on gender identity in society, and bridges the interesting narrative of human growth between childhood and adulthood (4).

### Impulsivity

Impulsivity been linked to pathological behaviours like rage outbursts, violence, suicidal or self-destructive activities, binge eating, social irresponsibility (5), extra marital affairs and marital adjustment (6, 7). Impulsivity can stem from difficulty in managing intense negative emotions accompanied by lack of rational thinking, frustration and poor problem solving often resulting in risky behaviours (8). Conversely, positive emotions (excitement, elation, adrenal rush) during adolescence may also increase the propensity for risky behaviours (9), highlighting the influence of mood on impulsivity. Recent researches, articulated motivational volitional model of suicidal behaviour (10), suggests that individuals with high impulsivity are more likely to progress from the stage of contemplating self-injury to the stage of executing self-harm (11). Thus, considering an individual’s current mood is crucial when studying impulsivity in the context of non-suicidal self-injury (NSSI).

### Non-Suicidal Self Injurious Behaviours

NSSI serves as a means of emotional expression and stress management among adolescents (12). Typically manifested during mid-adolescence, with heightened susceptibility in emerging adulthood (ages 18 years old–24 years old) (13), NSSI is utilised by individuals to cope with trauma, seek attention and to signal their need for assistance. Impulsivity as it predisposes adolescents to employ self-harm as a coping mechanism during emotional turmoil (14).

### Impulsivity and NSSI

Impulsive behaviours often involve taking risks without considering the potential consequences (15). Adolescents often engage in impulsive behaviours which may lead to negative outcomes, which can contribute to feelings of distress and, in turn, increase the likelihood of NSSI (14). Engaging in impulsive behaviours like impulsive buying can escalate the level of impulsivity in an individual (16). It is significant to address the pressing issue of impulsivity in a more critical context, particularly concerning NSSI among adolescents. Adolescents, in particular, are vulnerable to impulsive behaviour and understanding the link between impulsivity and NSSI is important.

### Biochemistry

The period between 14 years old and 20 years old of age is characterised by heightened susceptibility to intense mood fluctuations (17). During this stage, individuals can experience rapid shifts between elevated emotional states and heightened sensitivity to perceived slights. This period is considered vulnerable due to the heightened risk resulting from inadvertent actions. In adolescents, there is a pronounced instability in neurotransmitter levels, particularly in those with propensities for emotional dysregulation (18). This instability in neurotransmitter levels contributes to disruptive mood fluctuations, which, in turn, can lead towards self-harming or self-mutilating behaviours (19).

A study by Larsen et al. (20) which focused on emotion regulation in adolescents, found that those with compromised emotional regulation tend to have heightened emotional responses to external stimuli. Specifically, it observed that adolescents with difficulties in emotion regulation often exhibit rapid and intense fluctuations in neurotransmitter activity, particularly in response to emotional triggers. These fluctuations can contribute to mood disturbances. This reaction could manifest as externalising behaviours including: rage, aggressive behaviours such as throwing objects, head banging or self-inflicted hair pulling. Such behavioural responses could potentially stem from reduced serotonin levels during the episode (21). Serotonin, one of the neurotransmitters, is responsible for regulating mood, impulse control and emotional processing (22). An identified genetic investigation demonstrated that adolescents harbouring a minimum of one short allele within the serotonin transporter-linked polymorphic region (5-HTTLPR) of the SLC6A4 gene exhibited an elevated propensity for engaging in NSSI following exposure to pronounced interpersonal stressors (23).

Another neurotransmitter is responsible for impulsive behaviours that could lead towards NSSI. Glutamate is a major excitatory neurotransmitter in the brain and plays a key role in various neural processes, including impulsivity and emotional regulation (24). Imbalances in the glutamate system have been implicated in several psychiatric disorders, including mood disorders, aggression and self-injurious behaviours (25).

Dopamine, a neurotransmitter implicated in reward processing, motivation and emotional regulation, has been studied in relation to impulsivity (26), which is often a prominent factor in NSSI behaviours among adolescents. Research study by Blasco-Fontecilla et al., (27) suggests that alterations in dopamine neurotransmission might contribute to impulsivity, increasing the likelihood of engaging in risky or self-destructive behaviours like NSSI. Dysregulation in the dopamine system could lead to difficulties in assessing the potential consequences of one’s own actions, impairing decision-making and inhibition control (28). This can heighten impulsive tendencies, potentially making adolescents more susceptible to NSSI as a maladaptive coping mechanism (27)

A study by Plener et al. (29) investigated that apart from neurotransmitters, a few parts of the brain are also responsible for regulating impulses and in the assessment of consequences. The prefrontal cortex, particularly the ventromedial prefrontal cortex (vmPFC) and the dorsolateral prefrontal cortex (dlPFC), helps regulate impulses and assess the consequences of actions. When these areas are not functioning properly, individuals might struggle to inhibit impulsive behaviours. The prefrontal cortex, specifically the medial prefrontal cortex (mPFC), is involved in emotional regulation and processing. When this region is disrupted, emotional responses can become dysregulated, potentially leading to NSSI as a way to cope with intense emotions.

A study using brain scans (fMRI) by Brañas et al. (30) looked at how the emotional part of the brain works differently in teenagers who engage in NSSI. Research group discovered that these teenagers with NSSI and depression reacted more strongly to situations where they felt excluded, and the areas that were associated with feeling relief and reward showed dysregulation upon exclusion. The study found that when these teenagers saw emotional things, their brain reacted in a changed way, especially in areas related to feelings and thinking (medial prefrontal cortex and ventrolateral prefrontal cortex) (31).

## Conclusion

### Summary Table of the Findings

[Table t1-18mjms3104_sc]

**Table t1-18mjms3104_sc:** 

Neurotransmitters		
Serotonin	Glutamate	Dopamine
**Brain Anatomy**		
Prefrontal cortex (ventromedial prefrontal cortex, dorsolateral prefrontal cortex)	Medial prefrontal cortex	Ventrolateral prefrontal cortex

This commentary delves into the biochemical aspect of NSSI and impulsivity. Impulsivity and NSSI, which have been termed as problematic behaviours by society, only emphasises these behaviours as a result of personality traits. This in turn stigmatises these behaviours which prevents affected adolescents from getting help. If the masses could easily understand the biological and neurological processes behind this phenomenon, it would help them in understanding the individuals. This in turn would lead towards less stigmatisation and more helping behaviours towards adolescents with NSSI and impulsivity. The research work on biomarkers serves a stepping stone for future researches on this current matter, as it will help future researchers to further expand on these researches to find a solution of these issues among adolescents.
